# Association between insulin administration method and six-month neurological outcome in survivors of out-of-hospital cardiac arrest who underwent targeted temperature management

**DOI:** 10.1371/journal.pone.0279776

**Published:** 2022-12-30

**Authors:** Dong Hun Lee, Byung Kook Lee, Yong Soo Cho, Yong Hun Jung, Hyoung Youn Lee, Kyung Woon Jeung, Chun Song Youn, Soo Hyun Kim, on behalf of Korean Hypothermia Network Investigators

**Affiliations:** 1 Department of Emergency Medicine, Chonnam National University Hospital, Gwangju, Republic of Korea; 2 Department of Emergency Medicine, Chonnam National University Medical School, Gwangju, Republic of Korea; 3 Trauma center, Chonnam National University Hospital, Gwangju, Republic of Korea; 4 Department of Emergency Medicine, College of Medicine, The Catholic University of Korea, Seoul, Republic of Korea; Fondazione IRCCS Policlinico San Matteo, ITALY

## Abstract

We investigated the association of insulin administration method with the achievement of mean glucose ≤ 180 mg/dL and neurological outcomes in out-of-hospital cardiac arrest (OHCA) survivors who had hyperglycemia after the return of spontaneous circulation. From a multicenter prospective registry, we extracted the data of adult OHCA survivors who underwent targeted temperature management (TTM) between 2015 and 2018. Blood glucose levels every 4 h after initiating TTM were obtained for 72 h. We divided insulin administration methods into three categories: subcutaneous (SQI), intravenous bolus (IBI), and continuous intravenous (CII). We calculated the mean glucose and standard deviation (SD) of glucose. The primary outcome was the achievement of mean glucose ≤ 180 mg/dL. The secondary outcomes were the 6-month neurological outcome based on the Cerebral Performance Category (CPC) scale (good, CPC 1–2; poor, CPC 3–5), mean glucose, and SD of glucose. Of the 549 patients, 296 (53.9%) achieved mean glucose ≤ 180 mg/dL, and 438 (79.8%) had poor neurological outcomes, 134 (24.4%), 132 (24.0), and 283 (51.5%) were in the SQI, IBI, and CII groups, respectively. The SQI (adjusted odds ratio [aOR], 0.848; 95% confidence intervals [CIs], 0.493–1.461) and IBI (aOR, 0.673; 95% CIs, 0.415–1.091) groups were not associated with mean glucose ≤ 180 mg/dL and the SQI (aOR, 0.660; 95% CIs, 0.335–1.301) and IBI (aOR, 1.757; 95% CIs, 0.867–3.560) groups were not associated with poor neurological outcomes compared to the CII group. The CII (168 mg/dL [147–202]) group had the lowest mean glucose than the SQI (181 mg/dL [156–218]) and IBI (184 mg/dL [162–216]) groups. The CII (45.0[33.9–63.5]) group had a lower SD of glucose than the IBI (50.8 [39.1–72.0]) group. The insulin administration method was not associated with achieving mean glucose ≤ 180 mg/dL and 6-month neurological outcomes.

## Introduction

Cardiac arrest leads to various metabolic derangements due to ischemic-reperfusion injury even after the return of spontaneous circulation (ROSC). Hyperglycemia is one of the common abnormalities following cardiac arrest [1]. A well-known scientific observation is the association between hyperglycemia and poor neurological outcomes or increased mortality in cardiac arrest survivors [2–5]. Therefore, the target glucose level is generally recommended as < 180 mg/dL, this parameter is applied to critically ill patients [6], as in comatose cardiac arrest survivors, as protection against potential neurological injury, even if the ultimate target glycemic range has not been elucidated [7, 8].

In a historic comparison study, although the target glucose range of the continuous intravenous insulin (CII) method changed from conventional range (150 to 200 mg/dL) to intensive range (100 to 150 mg/dL) in patients with coronary artery bypass graft, the CII method has been proven to reduce mortality compared to the subcutaneous insulin (SQI) method [9]. Therefore, intravenous insulin infusion is the preferred route for administering insulin in critically ill patients [10]. A randomized controlled trial has revealed that conventional glucose control (< 180 mg/dL) was associated with lower mortality than intensive glucose control (81 to 108 mg/dL) in critically ill patients [6]. However, a randomized controlled trial that compared strict and conventional glucose control in comatose cardiac arrest survivors failed to find the optimal target glucose range [11]. Furthermore, although insulin is administrated subcutaneously or intravenously (bolus or continuous), the clinical difference according to the insulin administration in cardiac arrest survivors has not been investigated.

To address this question, we hypothesized that the insulin administration method would be related to blood glucose levels and, thus, to neurological outcomes. To examine this hypothesis, we used a multicenter registry of out-of-hospital cardiac arrest (OHCA) who underwent targeted temperature management (TTM) with blood glucose recordings for 72 h after the initiation of TTM.

## Materials and methods

### Study design and population

The Korean Hypothermia Network Prospective Registry (KORHN-PRO) has been gathering data on comatose adult (age ≥ 18 years) OHCA survivors who underwent TTM at 20 participating hospitals since October 2015 (KORHN-PRO; NCT02827422) [12]. The KORHN-PRO collects data on blood glucose after ROSC and every 4 h from the initiation of TTM to 72 h. We performed a retrospective analysis of the KORHN-PRO data between October 2015 and December 2018. The ethics and institutional review board of all participating hospitals approved KORHN-PRO. Written informed consent was obtained from all patients or patients’ proxies per national requirements and the principle of the Declaration of Helsinki [13]. Independent researchers assessed the neurological outcomes at 1 and 6 months after ROSC and recorded these as the Cerebral Performance Category (CPC) scale [14].

We included adult OHCA survivors who had hyperglycemia (> 180 mg/dL) within 24 h following ROSC. We excluded patients who had: insufficient data on glucose (glucose measurement less than six times during the 72 h after the initiation of TTM); no insulin for glucose control; no data on insulin treatment; no hyperglycemia within 24 h following ROSC; no data on 6-month neurological outcomes; died within 24 h after ROSC.

### TTM and glucose control

After ROSC, the target temperature range of 33–36°C was achieved as soon as possible. Sedatives were administered, and if needed, neuromuscular blockade to control shivering. Patients were rewarmed after completing the maintenance phase at 0.2–0.5°C/h. Sedatives and neuromuscular blockade were discontinued as the patient achieved normothermia. Blood glucose level was monitored and controlled to avoid hyperglycemia or hypoglycemia throughout post-cardiac arrest care following ROSC. Hyperglycemia was managed with SQI, intravenous bolus insulin (IBI), or CII according to each hospital protocol and the attending physician (S1 File). Moderate (< 70 mg/dL) or severe hypoglycemia (< 40 mg/dL) was managed with glucose-containing solution.

### Data collection

We extracted the following data from the KORHN-PRO: age; sex; body mass index (BMI); pre-existing illness; a witness of collapse; bystander cardiopulmonary resuscitation (CPR); first monitored rhythm (shockable or non-shockable); etiology of cardiac arrest (cardiac or non-cardiac); time from collapse to ROSC; epinephrine dose; serum lactate level after ROSC, PaO_2_ and PaCO_2_ after ROSC; glucose levels after ROSC and every 4 h during the 72 h from the initiation of TTM; sequential organ failure assessment (SOFA) within the first day following ROSC [15]; target temperature (33–34°C or 35–36°C); insulin administration method (SQI, IBI, or CII); CPC 6-months after ROSC.

The primary outcome was the achievement of mean blood glucose ≤ 180 mg/dL. The secondary outcomes were neurological outcomes assessed using CPC 6 months after ROSC, maximum glucose, mean glucose, a standard deviation (SD) of glucose, minimum glucose, moderate hypoglycemia, and severe hypoglycemia. The neurological outcomes were defined as good (CPC 1 or 2) or poor (CPC 3–5).

### Statistical analysis

We report continuous variables as median with interquartile ranges because all continuous variables had a non-normal distribution and categorical variables as the frequency with percentile. We used chi-square or Fisher’s exact test to compare categorical variables, as appropriate. We used the Mann–Whitney *U* test to compare continuous variables between two groups and the Kruskal–Wallis test to compare continuous variables among three groups. We performed posthoc analysis using a pair-wise Mann–Whitney *U* test with Bonferroni correction. We conducted logistic regression analyses to investigate the association between the insulin administration method and the achievement of mean blood glucose ≤ 180 mg/dL and the association between insulin administration methods and neurological outcomes. We selected the variables with a *p*-value < 0.05 in comparisons among insulin administration methods as covariates for the association between insulin administration methods and mean blood glucose ≤ 180 mg/dL. Additionally, we selected the covariates after performing the multivariable logistic regression analysis with the variables with a p-value < 0.2 in comparisons between groups of mean glucose ≤ 180 mg/dL and > 180 mg/dL. We also selected the covariates for the association between insulin administration methods and neurological outcomes through the multivariable logistic regression analysis with variables with a *p*-value < 0.2 in comparisons between neurological outcome groups. We selected the variables with a *p*-value < 0.05 in the multivariate logistic regression analyses as final covariates. We performed the Hosmer–Lemeshow test to test the goodness-of-fit of the logistic model. We also performed the multivariate logistic regression analysis to examine the association between glucose variables and neurological outcomes after adjusting covariates. We report the logistic regression analysis results as an adjusted odds ratio (aOR) with 95% confidence interval (CIs). We used IBM SPSS Statistics 26.0 for Windows (IBM Corp., Armonk, NY). A two-sided *p*-value < 0.05 was used to indicate statistical significance.

## Results

### Study population

**Fig 1:**
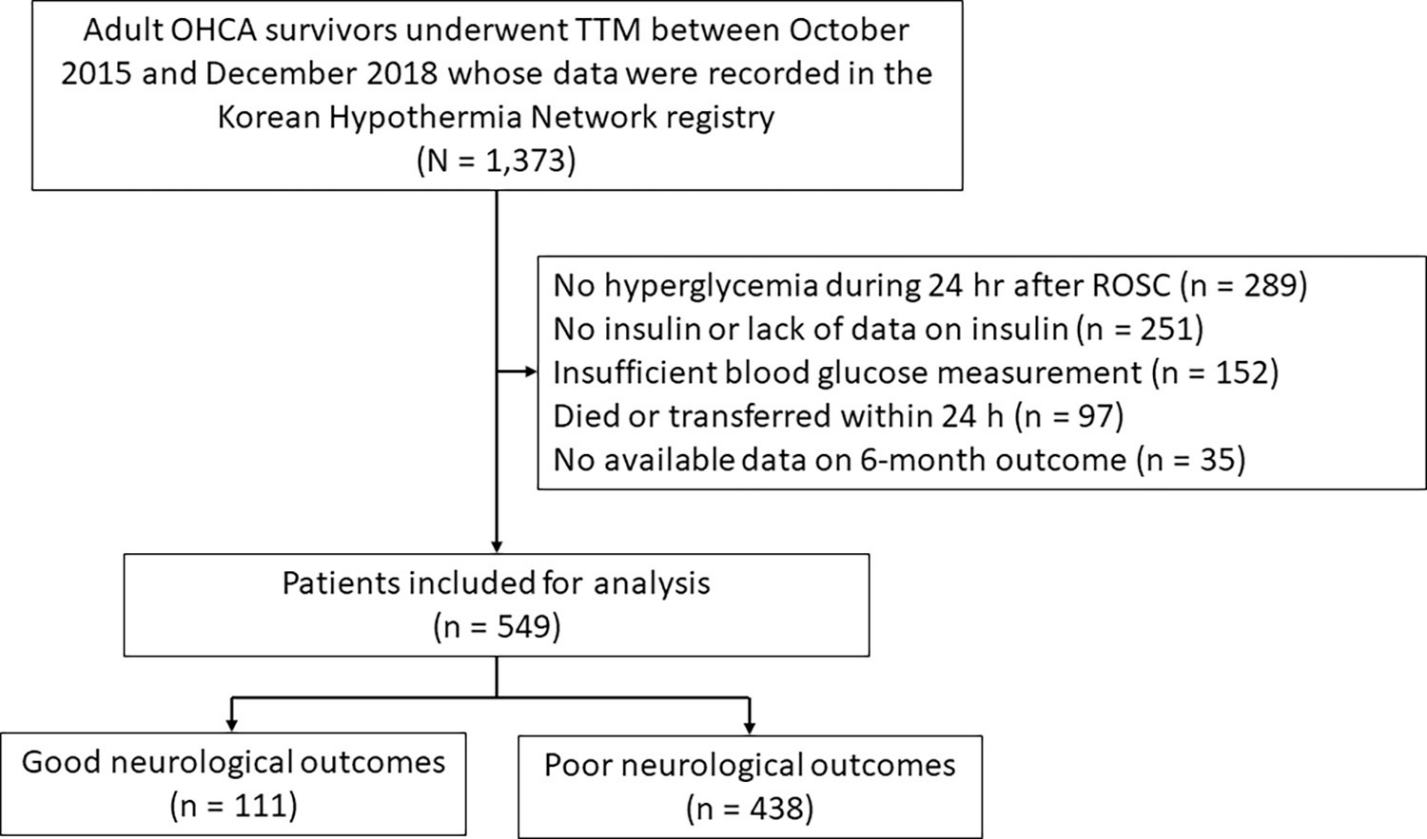
Flow chart describing the patient selection. OHCA, out-of-hospital cardiac arrest; TTM, targeted temperature management; ROSC, return of spontaneous circulation.

Of 1,373 OHCA survivors who were recorded in the registry, 289 patients had no hyperglycemia during 24 h after ROSC; 251 patients were either not administered insulin or lacked insulin data; 152 patients had insufficient blood glucose measurement (< 6 times) data during the 72 h after ROSC; 97 patients died or transferred within 24 h; 35 patients had no available data on 6-month CPC. Finally, 549 patients were included in the study (Fig 1).

### Characteristics according to the insulin administration method

Table 1 shows the baseline and clinical characteristics stratified by the insulin administration method. The SQI, IBI, and CII groups comprised 134 (24.4%), 132 (24.0%), and 283 (51.5%) patients, respectively. Pre-existing diabetes mellitus and renal disease, a witness of collapse, and etiology of cardiac arrest were different among the three groups. Time from collapse to ROSC and PaCO_2_ were different among the three groups. However, subgroup analyses found no difference in time from collapse to ROSC and PaCO_2_ between paired two groups. Pre-TTM shock and target temperature were different among the three groups.

**Table 1:** Baseline and clinical characteristics stratified by the insulin administration method.

Variables	SQI (n = 134)	IBI (n = 132)	CII (n = 283)	*p*
Age, years	60.0 (51.8–71.0)	60.0 (49.0–70.0)	61.0 (51.0–71.0)	0.769
Male sex	82 (61.2)	97 (73.5)	190 (67.1)	0.102
BMI, kg/m^2^	23.0 (20.8–25.4)	23.8 (20.2–25.9)	23.5 (21.1–26.0)	0.652
Pre-existing illness				
CAD	15 (11.2)	21 (15.9)	33 (11.7)	0.410
Heart failure	7 (5.2)	9 (6.8)	9 (3.2)	0.232
Hypertension	56 (41.8)	53 (40.2)	133 (47.0)	0.352
Diabetes mellitus	38 (28.4)	59 (44.7)	98 (34.6)	0.019
Stroke or TIA	14 (10.4)	12 (9.1)	15 (5.3)	0.126
Pulmonary disease	7 (5.2)	13 (9.8)	21 (7.4)	0.357
Renal disease	7 (5.2)	19 (14.4)	19 (6.7)	0.010
Liver cirrhosis	0 (0.0)	1 (0.8)	4 (1.4)	0.357
Witnessed	67 (50.0)	91 (68.9)	204 (72.1)	<0.001
Bystander CPR	78 (58.2)	86 (65.2)	174 (62.4)	0.508
Shockable rhythm	42 (31.3)	36 (27.3)	85 (30.0)	0.755
Cardiac etiology	72 (53.7)	83 (62.9)	190 (67.1)	0.030
Time to ROSC, min	37.0 (21.0–49.0)^a^	30.0 (19.0–43.0)^a^	32.0 (18.0–45.0)^a^	0.039
Epinephrine dose, mg	2 (1–5), 130	2 (1–4), 129	2 (1–4), 266	0.968
Serum lactate, mg/dL	10.3 (7.0–12.9)	10.7 (7.0–14.0)	9.9 (6.3–13.0)	0.283
Glucose affter ROSC, mg/dL	292 (213–356)	288 (228–372)	285 (231–355)	0.855
PaO_2,_ mmHg	102.1 (70.0–193.0)	107.0 (74.4–180.8)	109.3 (73.0–196.0)	0.407
PaCO_2,_ mmHg	45.1 (33.9–61.8)^a^	53.0 (39.0–75.9)^a^	50.0 (37.4–72.7)^a^	0.040
Pre-TTM shock	94 (70.1)	89 (67.4)	148 (52.3)	<0.001
SOFA	12 (10–13)	12 (10–13)	11 (9–13)	0.141
Target temperature				
33°C–34°C	63 (47.0)	114 (86.4)	270 (95.4)	<0.001
35°C–36°C	71 (53.0)	18 (13.6)	13 (4.6)	

SQI, subcutaneous insulin; IBI, intravenous bolus insulin; CII, continuous intravenous insulin; BMI, body mass index; CAD, coronary artery disease; TIA, transient ischemic attack; CPR, cardiopulmonary resuscitation; ROSC, return of spontaneous circulation; TTM, targeted temperature management; SOFA, sequential organ failure assessment

^a^Post-hoc analysis by pair-wise Mann–Whitney U test with Bonferroni correction showed no difference.

Table 2 shows the glucose variables according to the insulin administration method. Achievement of mean glucose ≤ 180 mg/dL, mean glucose, SD of glucose, and minimum glucose differed among the three groups. The CII group (168 mg/dL [147–202]) had lower mean glucose levels than the SQI (181 mg/dL [156–218]) and IBI (184 mg/dL [162–216]) groups. The IBI group (50.8 [39.1–72.0]) had higher SDs of glucose than the SQI (47.0 [33.2–61.1]) and CII (45.0 [33.9–63.5]) groups. The CII group (98 mg/dL [82–112]) had lower minimum glucose levels than the SQI group (106 mg/dL [91–122]). However, the IBI group had similar minimum glucose levels as the SQI and CII groups. Severe hypoglycemia was different among the three groups.

**Table 2:** Glucose characteristics during 72 h after cardiac arrest according to the insulin administration method.

Characteristics	SQI (n = 134)	IBI (n = 132)	CII (n = 283)	*p*
MG ≤ 180 mg/dL	65 (48.5)	61 (46.2)	170 (60.1)	0.011
Mean, mg/dL	181 (156–218)^a^	184 (162–216)^a^	168 (147–202)^b^	0.001
Maximum, mg/dL	338 (269–415)	357 (285–417)	325 (268–389)	0.080
SD	47.0 (33.2–61.1)^a^	50.8 (39.1–72.0)^b^	45.0 (33.9–63.5)^a^	0.012
Minimum, mg/dL	106 (91–122)^a^	101 (82–114)^a^^,b^	98 (82–112)^b^	0.008
Moderate hypoglycemia	11 (8.2)	13 (9.8)	34 (12.0)	0.475
Severe hypoglycemia	0 (0.0)	8 (6.1)	9 (3.2)	0.017

SQI, subcutaneous insulin; IBI, intravenous bolus insulin; CII, continuous intravenous insulin; MG, mean glucose; SD, standard deviation

^a,b^Post-hoc analysis by pair-wise Mann–Whitney U test with Bonferroni correction. The same letter means no difference.

### Association between insulin administration method and mean glucose under 180 mg/dL

Table 3 shows the demographic and clinical characteristics stratified by mean glucose of 180 mg/dL. A total of 296 (53.9%) achieved mean glucose ≤ 180 mg/dL. Patients who had mean glucose ≤ 180 mg/dL had lower BMI (23.0 kg/m^2^ [20.7–25.4]) vs. 23.9 kg/m^2^ [21.3–26.1]) than those had mean glucose > 180 mg/dL. Patients with mean glucose ≤ 180 mg/dL had fewer pre-existing illnesses (hypertension and diabetes mellitus). Patient who had mean glucose ≤ 180 mg/dL had fewer witnesses of collapse (208/296 vs. 154/253), lower epinephrine dose (2 mg [0–4] vs. 2 mg [1–5]), lower serum lactate (9.3 mg/dL [6.0–12.6] vs. 11.3 mg/dL [8.0–14.0]), higher PaO_2_ (117.5 mmHg [76.0–221.0] vs. 100.0 mmHg [71.0–160.0]), lower pre-TTM shock (155/296 vs. 176/253), lower SOFA scores (11 [9–13] vs. 12 [10–14]), and a higher proportion of target temperature of 33°C–34°C (255/296 vs. 192/253).

**Table 3:** Demographic and clinical characteristics stratified by mean glucose of 180 mg/dL.

Variables	Total (n = 549)	MG ≤ 180 mg/dL (n = 296)	MG > 180 mg/dL (n = 253)	*p*
Age, years	61.0 (50.0–71.0)	59.0 (49.0–71.0)	62.0 (53.5–71.0)	0.093
Male sex	369 (67.2)	199 (67.2)	170 (67.2)	1.000
Body mass index, kg/m^2^	23.5 (20.9–25.8)	23.0 (20.7–25.4)	23.9 (21.3–26.1)	0.025
Pre-existing illness				
Coronary artery disease	69 (12.6)	31 (10.5)	38 (15.0)	0.141
Heart failure	25 (4.6)	12 (4.1)	13 (5.1)	0.688
Hypertension	242 (44.1)	112 (37.8)	130 (51.4)	0.002
Diabetes mellitus	195 (35.5)	63 (21.3)	132 (52.2)	<0.001
Stroke or TIA	41 (7.5)	18 (6.1)	23 (9.1)	0.240
Pulmonary disease	41 (7.5)	28 (9.5)	13 (5.1)	0.079
Renal disease	45 (8.2)	25 (8.4)	20 (7.9)	0.941
Liver cirrhosis	5 (0.9)	2 (0.7)	3 (1.2)	0.860
Witnessed	362 (65.9)	208 (70.3)	154 (60.9)	0.026
Bystander CPR	338 (61.6)	185 (62.5)	153 (60.5)	0.690
Shockable rhythm	163 (29.7)	98 (33.1)	65 (25.7)	0.072
Cardiac etiology	345 (62.8)	184 (62.2)	161 (63.6)	0.789
Time to ROSC, min	32.0 (19.0–45.0)	32.0 (19.0–45.0)	33.0 (19.0–46.0)	0.365
Epinephrine dose, mg	2 (1–4)	2 (0–4)	2 (1–5)	0.017
Serum lactate, mg/dL	10.2 (6.7–13.3)	9.3 (6.0–12.6)	11.3 (8.0–14.0)	<0.001
Glucose after ROSC, mg/dL	287 (228–360)	263 (215–323)	323 (242–409)	<0.001
PaO_2,_ mmHg	106.8 (73.0–191.0)	117.5 (76.0–221.0)	100.0 (71.0–160.0)	0.001
PaCO_2,_ mmHg	49.6 (37.0–71.4)	48.3 (37.1–69.5)	54.0 (38.8–75.0)	0.343
Pre-TTM shock	331 (60.3)	155 (52.4)	176 (69.6)	<0.001
SOFA	12 (9–13)	11 (9–13)	12 (10–14)	<0.001
Target temperature				0.003
33°C–34°C	447 (81.4)	255 (86.1)	192 (75.9)	
35°C–36°C	102 (18.6)	41 (13.9)	61 (24.1)	

MG, mean glucose; TIA, transient ischemic attack; CPR, cardiopulmonary resuscitation; ROSC, return of spontaneous circulation; TTM, targeted temperature management; SOFA, sequential organ failure assessment

Multivariable logistic regression analysis found hypertension (aOR, 0.609; 95% CIs, 0.399–0.930), diabetes mellitus (aOR, 0.273; 95% CIs, 0.175–0.425), witnessed (aOR, 1.992; 95% CIs, 1.289–3.078), PaO_2_ (aOR, 1.002; 95% CIs, 1.000–1.004), pre-TTM shock (aOR, 0.541; 95% CIs, 0.355–0.826), target temperature of 35–36°C (aOR, 0.500; 95% CIs, 0.297–0.842), glucose after ROSC (aOR, 0.995; 95% CIs, 0.993–0.997) as covariates. The insulin administration method was not associated with the achievement of mean glucose ≤ 180 mg/dL after adjusting covariates (Table 4).

**Table 4:** The association between insulin administration method and mean glucose ≤ 180 mg/dL.

Variables	Adjusted odds ratio (95% confidence interval)	*p*
Hypertension	0.686 (0.458–1.027)	0.067
Diabetes mellitus	0.274 (0.180–0.417)	< 0.001
Renal disease	1.559 (0.742–3.277)	0.241
Witnessed	1.752 (1.156–2.656)	0.008
Cardiac etiology	0.891 (0.575–1.381)	0.607
Time to ROSC	0.999 (0.989–1.009)	0.820
Glucose after ROSC, mg/dL	0.995 (0.993–0.997)	<0.001
PaO_2,_ mmHg	1.003 (1.001–1.004)	
PaCO_2,_ mmHg	1.003 (0.996–1.011)	0.387
Pre-TTM shock	0.580 (0.389–0.864)	0.007
Target temperature		
33°C–34°C	Reference	
35°C–36°C	1.819 (1.094–3.025)	0.021
Insulin administration method		
CII	Reference	
SQI	0.848 (0.493–1.461)	0.553
IBI	0.673 (0.415–1.091)	0.108

ROSC, return of spontaneous circulation; TTM, targeted temperature management; CII, continuous intravenous insulin; SQI, subcutaneous insulin; IBI, intravenous bolus insulin

### Association between insulin administration method and neurological outcomes

Table 5 shows the demographic and clinical characteristics according to the neurological outcomes. A total of 438 (79.8%) patients had poor neurological outcomes. Patients in the poor neurological outcome group were older (62.0 years [51.8–72.0] vs. 58.0 years [48.0–67.0]) than those in the good neurological outcome group, although BMI and pre-existing illness were not different between neurological outcome groups. Those in the poor neurological outcome group had fewer witnesses of collapse (268/438 vs. 94/111), a lower proportion of bystander CPR (256/438 vs. 82/111), a lower proportion of shockable rhythm (83/438 vs. 80/111), fewer cardiac etiology (242/438 vs. 103/111), longer time from collapse to ROSC (36.0 min [23.0–47.0] vs. 18.0 min [12.0–27.0]), and received higher epinephrine dose (2 mg [1–4] vs. 0 mg [0–2]) than those in the good neurological outcome group. Those in the poor neurological outcome group had higher serum lactate levels after ROSC (10.9 mg/dL [7.5–13.7] vs. 7.5 mg/dL [4.1–11.2]), higher PaCO_2_ (53.7 mmHg [39.0–75.1] vs. 39.0 mmHg [32.2–52.4]), more pre-TTM shock (282/438 vs. 49/111), and higher SOFA scores (12 [10–13] vs. 10 [8–12]).

**Table 5:** Demographic and clinical characteristics stratified by neurological outcomes.

Variables	Good (n = 111)	Poor (n = 438)	*p*
Age, years	58.0 (48.0–67.0)	62.0 (51.8–72.0)	0.006
Male sex	80 (72.1)	289 (66.0)	0.222
Body mass index, kg/m^2^	23.6 (21.5–25.3)	23.4 (20.8–26.0)	0.718
Pre-existing illness			
Coronary artery disease	20 (18.0)	49 (11.2)	0.052
Heart failure	5 (4.5)	20 (4.6)	0.978
Hypertension	47 (42.3)	195 (44.5)	0.680
Diabetes mellitus	34 (30.6)	161 (36.8)	0.228
Stroke or TIA	9 (8.1)	32 (7.3)	0.774
Pulmonary disease	6 (5.4)	35 (8.0)	0.355
Renal disease	5 (4.5)	40 (9.1)	0.112
Liver cirrhosis	1 (0.9)	4 (0.9)	1.000
Witnessed	94 (84.7)	268 (61.2)	<0.001
Bystander CPR	82 (73.9)	256 (58.4)	0.003
Shockable rhythm	80 (72.1)	83 (18.9)	<0.001
Cardiac etiology	103 (92.8)	242 (55.3)	<0.001
Time to ROSC, min	18.0 (12.0–27.0)	36.0 (23.0–47.0)	<0.001
Epinephrine dose, mg	0 (0–2)	2 (1–4)	<0.001
Serum lactate, mg/dL	7.5 (4.1–11.2)	10.9 (7.5–13.7)	<0.001
Glucose after ROSC, mg/dL	273 (214–334)	291 (229–368)	0.068
PaO_2,_ mmHg	94.0 (72.0–147.0)	111.0 (74.1–196.2)	0.028
PaCO_2,_ mmHg	39.0 (32.2–52.4)	53.7 (39.0–75.1)	<0.001
Pre-TTM shock	49 (44.1)	282 (64.4)	<0.001
SOFA	10 (8–12)	12 (10–13)	<0.001
Target temperature			0.865
33°C–34°C	91 (82.0)	356 (81.3)	
35°C–36°C	20 (18.0)	82 (18.7)	

TIA, transient ischemic attack; CPR, cardiopulmonary resuscitation; ROSC, return of spontaneous circulation; TTM, targeted temperature management; SOFA, sequential organ failure assessment

Multivariate logistic regression analysis found that age (aOR, 1.031; 95% CIs, 1.008–1.054), shockable rhythm (aOR, 0.183; 95% CIs, 0.096–0.349), cardiac etiology (aOR, 0.196; 95% CIs, 0.076–0.506), time from collapse to ROSC (aOR, 1.074; 95% CIs, 1.048–1.102), PaO_2_ (aOR, 1.003; 95% CIs, 1.000–1.006), PaCO_2_ (aOR, 1.015; 95% CIs, 1.001–1.029), and pre-TTM shock (aOR, 1.855; 95% CIs, 1.037–3.319) were associated with poor neurological outcome. The insulin administration method was not associated with the 6-month neurological outcomes after adjusting covariates (Table 6).

**Table 6:** The association between insulin administration method and poor neurological outcomes.

Variables	Adjusted odds ratio (95% confidence interval)	*p*
Age, years	1.037 (1.015–1.059)	0.001
Diabetes mellitus	0.792 (0.427–1.471)	0.460
Renal disease	1.469 (0.461–4.680)	0.515
Witnessed	0.776 (0.378–1.590)	0.488
Shockable rhythm	0.182 (0.099–0.336)	< 0.001
Cardiac etiology	0.203 (0.083–0.496)	< 0.001
Time to ROSC, min	1.063 (1.042–1.083)	< 0.001
PaO_2_, mmHg	1.003 (1.000–1.005)	0.049
PaCO_2_, mmHg	1.014 (1.001–1.028)	0.036
Pre-TTM shock	1.632 (0.933–2.854)	0.086
Target temperature	1.240 (0.533–2.882)	0.617
33°C–34°C	Reference	
35°C–36°C		
Insulin administration method		
CII	Reference	
SQI	0.660 (0.335–1.301)	0.230
IBI	1.757 (0.867–3.560)	0.118

ROSC, return of spontaneous circulation; TTM, targeted temperature management; CII, continuous intravenous insulin; SQI, subcutaneous insulin; IBI, intravenous bolus insulin

### Association between glucose variables and neurological outcomes

Table 7 shows the glucose variables stratified by neurological outcome groups. The poor neurological outcome group had higher maximum glucose (343 mg/dL [284–410] vs. 308 mg/dL [250–366]), mean glucose (179 mg/dL [154–216] vs. 164 mg/dL [145–189]), SD (48.2 [35.9–67.0] vs. 41.1 [29.9–52.9]), and more frequent moderate hypoglycemia (52/438 vs. 6/111) than the good neurological outcome group (Table 7).

**Table 7:** Glucose characteristics during 72 h after cardiac arrest stratified by neurological outcomes.

Characteristics	Good (n = 111)	Poor (n = 438)	*p*
Maximum, mg/dL	308 (250–366)	343 (284–410)	<0.001
Mean, mg/dL	164 (145–189)	179 (154–216)	<0.001
SD	41.1 (29.9–52.9)	48.2 (35.9–67.0)	<0.001
Minimum, mg/dL	98 (89–111)	100 (82–116)	0.590
Moderate hypoglycemia	6 (5.4)	52 (11.9)	0.048
Severe hypoglycemia	4 (3.6)	13 (3.0)	0.730

SD, standard deviation

Table 8 shows the association between glucose variables during 72 h and neurological outcomes. Maximum glucose (aOR, 1.004; 95% CIs, 1.001–1.007), mean glucose (aOR, 1.009; 95% CIs, 1.002–1.016), andSD (aOR, 1.019; 95% CIs, 1.005–1.032) of glucose were independently associated with poor neurological outcomes.

**Table 8:** The association between glucose variables during 72 h and neurological outcomes.

Characteristics	Adjusted odds ratio (95% confidence interval)	*p*
Maximum	1.004 (1.001–1.007)	0.004
Mean	1.009 (1.002–1.016)	0.013
SD	1.019 (1.005–1.032)	0.006
Minimum	1.002 (0.993–1.011)	0.688
Moderate hypoglycemia	1.331 (0.457–3.878)	0.600
Severe hypoglycemia	0.856 (0.176–4.162)	0.847

SD, standard deviation

Adjusted for age, first monitored rhythm, etiology of cardiac arrest, time from collapse to ROSC, PaO_2_ after ROSC_,_ PaCO_2_ after ROSC, and pre-targeted temperature management shock

## Discussion

This retrospective analysis found that the insulin administration method had no association with the achievement of mean glucose ≤ 180 mg/dL and the 6-month neurological outcome. Nevertheless, the CII group had the lowest mean glucose levels, and the IBI group had the highest SD of glucose levels. The CII and IBI groups had lower minimum glucose levels than the SQI group. The IBI group had a higher incidence of severe hypoglycemia. Higher maximum glucose, mean glucose, and SD of glucose were independently associated with poor neurological outcomes.

CII is the preferred route and delivery method of insulin in critically ill patients with hyperglycemic crises, such as perioperative care of cardiac surgery, cardiogenic shock, myocardial infarction, and acute ischemic stroke [16]. Such situations may require a rapid change in the insulin level or be associated with poor perfusion of subcutaneous tissue; for these reasons, guidelines recommend CII in critically ill patients instead of SQI [17]. A study comparing CII to SQI in patients with diabetes undergoing coronary artery bypass grafting demonstrated that CII was associated with lower postoperative blood glucose and reduced mortality [9]. Nevertheless, SQI is still used to control blood glucose in critically ill patients [18]. Likewise, IBI has a comparable effect on blood glucose to CII without any adverse effect [19]. Although CII was used the most, SQI and IBI were also used in about half of the patients in the present study. We found that the SQI and IBI groups had higher mean blood glucose levels during the 72 h after TTM initiation than the CII group. Higher mean blood glucose level was associated with the 6-month poor neurological outcomes in the present study. However, SQI or IBI had no association with the achievement of mean glucose ≤ 180 mg/dL and the 6-month neurological outcomes compared to CII. It might be postulated that SQI or IBI is not inferior to CII regarding controlling hyperglycemia, considering the independent association between high mean glucose and poor neurological outcome.

Regardless of the relationship of the insulin administration method with clinical outcomes, regarding glycemic control, CII seems to be the best measure among the three insulin administration methods. Hyperglycemia early after ROSC, at admission, or during 36 h after admission in survivors of cardiac arrest who underwent therapeutic hypothermia or TTM was associated with poor neurological outcomes [2, 4, 5]. The blood glucose level after ROSC was not associated with the neurological outcomes in the present study. We postulate that because we excluded the patients without hyperglycemia within 24 h after ROSC, it might affect the insignificant relationship between glucose after ROSC and neurological outcomes; this is because the patients with no hyperglycemia after ROSC would be injured less than those with hyperglycemia due to ischemic insult. However, consistent with the previous study reports, high maximum and mean glucose levels during the 72 h following initiation of TTM were associated with poor neurological outcomes in the present study. Our results strengthen the importance of glucose control for a more extended period following ROSC.

Blood glucose level and glucose variability have been assessed in comatose cardiac arrest survivors, and high glucose variability is associated with poor neurological outcomes [2, 3, 5]. We calculated the SD of glucose for 72 h after the initiation of TTM to determine glucose variability. We also found that high SD of glucose was associated with poor neurological outcomes in the present study. Although there was no association between the insulin administration method and neurological outcomes, lower mean glucose levels and lower SD of glucose associated with favorable neurological outcomes emphasize that the CII may be the best measure to control glycemic status in cardiac arrest survivors.

A landmark trial regarding glucose control in critically ill patients revealed that intensive glucose control (81 to 110 mg/dL) increased mortality in the intensive care unit, and the high mortality in the intensive glucose control group was associated with iatrogenic hypoglycemia [6, 20]. Therefore, avoiding hypoglycemia is as crucial as avoiding hyperglycemia when controlling blood glucose. Although we found no association between neurological outcomes and moderate or severe hypoglycemia, the IBI and CII groups had frequent severe hypoglycemia compared to the SQI group in the present study. Frequent blood glucose monitoring is required more in the IBI and CII groups than in the SQI group to avoid hypoglycemia. Conventional glucose control (target ≤ 180 mg/dL) resulted in total 16.3% of hypoglycemia (moderate hypoglycemia, 15.8%; severe hypoglycemia, 0.5%) in a randomized control trial [6]. We reported 13.7% of hypoglycemia (moderate hypoglycemia, 10.6%; severe hypoglycemia, 3.1%). In the randomized control group, patients in the conventional glucose control group had a history of diabetes mellitus in about 20%, whereas we reported that 35.5% of the patients had a history of diabetes mellitus [6]. Diabetes mellitus does not seem to play a role in hypoglycemia during insulin treatment.

This study has several limitations. Although we used the data from the prospective multicenter registry, we can only demonstrate an association because this is a retrospective analysis. Of the total registry, about 60% of the patients were excluded, which might have caused selection bias. The registry is configured to record blood glucose every 4 h during TTM. Therefore, glucose characteristics might be different from the actual values. Although diabetes mellitus was not associated with neurological outcomes, pre-existing diabetes mellitus differed among the insulin treatment groups. Glycated hemoglobin has been reported to be associated with neurological outcomes in comatose cardiac arrest survivors [21], which means that glucose control status before cardiac arrest rather than the history of diabetes mellitus might contribute to neurological outcomes. However, we could not analyze the data according to glycated hemoglobin level due to the limitation of data. Future studies need to address the interaction of the insulin administration method with glycemic control status. The target temperature differed among the insulin treatment groups because the target temperature and the insulin administration method depended on the post-cardiac arrest care protocol at each hospital rather than the attending physician. However, the target temperature was not associated with neurological outcomes in the present study. The targeted temperature management trial also showed no difference in blood glucose and glucose variability between the target temperature groups [2]. Even between CII and IBI, the detailed administration method is different. The total insulin dosage may vary depending on the insulin administration method. Due to data limitations, it was impossible to compare the total amount of insulin administered in the present study.

## Conclusions

The insulin administration method was not associated with the achievement of mean glucose ≤ 180 mg/dL and the 6-month neurological outcomes in OHCA survivors who underwent TTM. The CII method had lower mean glucose and lower SD of glucose rather than the IBI and SQI methods.

## Supporting information

S1 File Insulin administration protocols. The participating hospitals in Korean Hypothermia Network have their insulin administration protocol. The protocols are a bit different from each other.
(PDF)

S1 Data Raw data. (XLSX)
