# Supplementary material for: Association between insulin administration method and six-month neurological outcome in survivors of out-of-hospital cardiac arrest who underwent targeted temperature management
Source: PLoS One. 2022 Dec 30;17(12):e0279776. doi: 10.1371/journal.pone.0279776 (PMC9803235; doi:10.1371/journal.pone.0279776)
Supplement: S1 File — The participating hospitals in Korean Hypothermia Network have their insulin administration protocol. The protocols are a bit different from each other. (PDF) [file pone.0279776.s001.pdf]

Continuous intravenous insulin #1

1. Initiating an insulin infusion

   Infusion mix: Regular insulin 50 U + normal saline 50 cc

**Blood glucose**	**Infusion rate (u/hr)**
181-220	1
221-260	2
261-300	3
≥ 301	4

1) Check the blood glucose every 2 hr until target glucose is achieved.

2) Check the blood glucose every 4 hr after achievement of target glucose

2. Changing the insulin infusion rate according to the difference in blood glucose

**Blood glucose (mg/dL)**	**Blood glucose change (mg/dL)**		**Infusion rate change***
	increase	decrease	
80-89			Stop infusion
90-99	≥ 0		Maintain infusion
		1-20	Reduce infusion rate
		≥ 21	Stop infusion
100-180	≥ 31		Increase infusion rate
	0 - 30	0 – 30	Maintain infusion
		31 – 50	Reduce infusion rate
		≥ 51	Stop infusion for 1hr and reduce infusion rate
181-220	≥ 1	0	Increase infusion rate
		1 – 40	Maintain infusion rate
		41 – 80	Reduce infusion rate
		≥ 81	Stop infusion 1hr and reduce
≥ 221	≥ 1	0	Double infusion rate
		1 – 40	Increase infusion rate
		41 – 80	Maintain infusion rate
		81 – 120	Reduce infusion rate
		≥ 121	Stop infusion for 1 hr and reduce infusion rate by 1/2

* Changes in infusion rate

Current infusion rate (u/hr)	Reduce or increase infusion rate
≤ 3	1
3.5 – 6	2
≥ 3.5	3

Continuous intravenous insulin #2

Markovitz insulin drip protocol

Goal BG =_______

General Guidelines:

- Standard drip 100 Units/100ml PNSS via an insulin infusion device.

ALGORITHM 1		ALGORITHM 2		ALGORITHM 3		ALGORITHM 4	
BG	Units/hr	BG	Units/hr	BG	Units/hr	BG	Units/Hr
< 70 = HYPOGLCEMIA (see below for treatment)							
<70	OFF	<70	Off	<70	Off	<70	Off
70-109	0.2	70-109	0.5	70-109	1	70-109	1.5
110-119	0.5	110-119	1	110-119	2	110-119	3
120-149	1	120-149	1.5	120-149	2	120-149	3
150-179	1.5	150-179	2	150-179	4	150-179	7
180-209	2	180-209	3	180-209	5	180-209	9
210-239	2	210-239	4	210-239	6	210-239	12
240-269	3	240-269	5	240-269	8	240-269	16
270-299	3	270-299	6	270-299	10	270-299	20
300-329	4	300-329	7	300-329	12	300-329	24
330-359	4	330-359	8	330-359	14	330-359	28
>360	6	>360	12	>360	16	>360	28

Moving from algorithm to algorithm

• **Move up to the next higher algorithm** if the blood glucose concentration is above the goal range (see above goal) and does not change by at least 60 mg/dL within 1 hour.
• **Move down an algorithm** when blood glucose is < 100 mg/dL X 2.

Patient monitoring
• Check capillary blood glucose every hour until it is within goal range for 4 hours, then decrease to every 2 hours for 4 hours, and if it remains stable, may decrease to every 4 hours
• Hourly monitoring may be indicated for critically ill patients even if they have stable blood glucose

Treatment of hypoglycemia (blood glucose < 70 mg/dL)
• Discontinue insulin drip AND
• Give dextrose 50% in water (D50W) intravenously
If patient is conscious: 25 mL (1/2 vial)
If patient is not conscious: 50 mL (1 vial)
• Recheck blood glucose every 15-20 minutes and repeat 25 mL of D50W IV if < 70 mg/dL. Restart insulin drip once blood glucose is > 140 mg/dL X 2 checks. Restart drip with lower algorithm (see "Moving down")

Notify the physician
• For any blood glucose change greater than 100 mg/dL in 1 hour
• For blood glucose > 360 mg/dL
• For hypoglycemia that has not resolved within 20 minutes of giving 50 mL of D50W IV and discontinuing the insulin drip

Appendix 2. Sample Size Computation: Comparing Two Proportions

Incidence of hypoglycemia for the physician directed protocol[7]	5.1%
Incidence of hypoglycemia for Markovitz protocol[7]	4%
**Sample size per group**	**412**

1. **Formula for sample size n**

$n = (Z_{\alpha/2}+Z_{\beta})^2 * (p_1(1-p_1)+p_2(1-p_2)) / (p_1-p_2)^2$,

where $Z_{\alpha/2}$ is the critical value of the Normal distribution at $\alpha/2$ (e.g. for a confidence level of 95%, α is 0.05 and the critical value is 1.96), Z_{β} is the critical value of the Normal distribution at β (e.g. for a power of 80%, β is 0.2 and the critical value is 0.84) and p_1 and p_2 are the expected sample proportions of the two groups.

Reference: Markovitz LJ, Wiechmann RJ, Harris N, Hayden V, Cooper J, Johnson G, Harelstad R, Calkins L, Braithwaite SS 2002 Description and evaluation of a glycemic management protocol for patients with diabetes undergoing surgery. Endocr Pract 8:10–18

Continuous intravenous insulin #3

**Continuous IV**	**Blood glucose**	**infusion rate**
RI 250 IU mixed in normal saline 500 cc	>450	25 cc/hr
	351 - 450	12 cc/hr
	301 - 350	10 cc/hr
	251 - 300	8 cc/hr
	201 - 250	6 cc/hr
	151 - 200	4 cc/hr
	≤150	stop

Continuous intravenous insulin #4

**Continuous IV**	**Blood glucose**	**infusion rate**
RI 250 IU mixed in normal saline 500 cc	>450	40 cc/hr
	351 - 450	10 cc/hr
	301 - 350	8 cc/hr
	251 - 300	6 cc/hr
	201 - 250	4 cc/hr

Bolus intravenous insulin #1

Bolus intravenous insulin	Blood glucose	insulin dose
	>400	12 U
	351 - 400	10 U
	301 - 350	8 U
	251 - 300	6 U
	201 - 250	4 U

Bolus intravenous insulin #2

Bolus intravenous insulin	Blood glucose	insulin dose
	>400	10 U
	351 - 400	8 U
	301 - 350	6 U
	251 - 300	4 U
	201 - 250	2 U

Bolus intravenous insulin #3

Bolus intravenous insulin	Blood glucose	insulin dose
	>400	6 IU
	351 - 400	6 IU
	301 - 350	4 IU
	251 - 300	4 IU
	201 - 250	2 IU

Subcutaneous insulin #1

**Subcutaneous insulin**	**BST**	**insulin dose**
	>300	6 IU
	251 - 300	5 IU
	201 - 250	4 IU
