# Supplementary material for: Association between insulin administration method and six-month neurological outcome in survivors of out-of-hospital cardiac arrest who underwent targeted temperature management
Source: PLoS One. 2022 Dec 30;17(12):e0279776. doi: 10.1371/journal.pone.0279776 (PMC9803235; doi:10.1371/journal.pone.0279776)
Supplement: Attachment — Submitted filename: response to reviewers.docx [file pone.0279776.s003.docx]

**Dear editor, we revised the manuscript according to the requirement of the journal.**

**Editor**

When submitting your revision, we need you to address these additional requirements.

1. Please ensure that your manuscript meets PLOS ONE's style requirements, including those for file naming.

Answer) We revised the manuscript according to the PLOS One style requirements.

2. Thank you for stating in your Funding Statement: 
"This study was supported by a grant from National Research Foundation of Korea (NRF-2021R1A2C1005800)."

Please provide an amended statement that declares *all* the funding or sources of support (whether external or internal to your organization) received during this study, as detailed online in our guide for authors at http://journals.plos.org/plosone/s/submit-now.  Please also include the statement “There was no additional external funding received for this study.” in your updated Funding Statement. 
Please include your amended Funding Statement within your cover letter. We will change the online submission form on your behalf.

Answer) We added the sentence as the editor advised in the Funding Statement.

3. In your Data Availability statement, you have not specified where the minimal data set underlying the results described in your manuscript can be found. PLOS defines a study's minimal data set as the underlying data used to reach the conclusions drawn in the manuscript and any additional data required to replicate the reported study findings in their entirety. All PLOS journals require that the minimal data set be made fully available. We will update your Data Availability statement to reflect the information you provide in your cover letter.

Answer) we added the minimal data set as a Supplementary file.

**Dear reviewers, we appreciate your kind review. They were very helpful in improving our manuscript. After due consideration, the manuscript was revised as described below.**

Response to Reviewers

Reviewer #1:

1. It would be interesting to know if there were statistical differences in terms of total unit of insulin administered between the three methods.

Answer) As you indicated, it would be interesting to know whether the amount of insulin administered varies depending on the insulin administration methods. We also thought that the topic would be very relevant. However, the registry was built to input insulin dose data optionally. Therefore, calculating insulin dose using our data is inappropriate because the input of insulin dose information was not completed, and insulin administered more frequently than every 4 hrs is not entered.

We added the following sentence in the limitation

“The total insulin dosage may vary depending on the insulin administration method. Due to data limitations, it was impossible to compare the total amount of insulin administered in the present study.”

2. At line 261 “A landmark trial regarding glucose control in critically ill patients revealed that intensive glucose control (< 180 mg/dL) increased mortality in the intensive care unit” is probably a typo since, according to the reference (6), the intensive glucose control in the trial was between 81 and 10mg/dl.

Answer) Thank you for finding the typo. We revised as “intensive glucose control (81 to 110 mg/dL)

Reviewer #2: Review PONE-D-22-17886

In this article authors retrospectively investigate the association between insulin administration method and neurological outcomes in out-of-hospital cardiac arrest (OHCA) survivors; data were extracted from a multicentre registry of patients with OHCA who underwent targeted temperature management (TTM). Authors ambitiously tried to use Cerebral Performance Category Scale as primary endpoint, evaluating its association with insulin administration method. Insulin regulating mechanism is known to be altered in patient with cardiac arrest. However, the physiopathological mechanism according to which it has been hypothesized that insulin administration method would be associated with neurological outcome is not clearly described: maybe one way of administration (CII) should better achieve the optimal glucose target? In this case, first step analysis should address this physiopathological mechanism; the association between insulin administration method and neurological outcomes in OHCA should be investigated secondarily.

Major issues

1. The three different groups according to way of insulin administration (SQI, IBI and CII) are characterized by significantly different glucose values, prevalence of diabetes mellitus and other parameters which are described to influence neurological outcome (witnesses to collapse, cardiac aetiology, time from collapse to ROSC). Based on above findings, the groups can't be considered homogeneous.

Answer) Considering the pathophysiologic mechanism, as suggested by the reviewer, the primary outcome was mean glucose ≤ 180 mg/dL. We added multivariate regression analysis, which investigated the association between the insulin administration method and mean glucose ≤ 180 mg/dL after adjusting the covariates.

To control the heterogenous characteristics among three insulin administration methods, variables that are different in the univariate analysis (comparison among insulin administration methods) and variables that are independently associated with mean glucose ≤ 180 mg/dL and neurological outcomes were also included in the multivariable logistic regression analysis. Therefore, we added two tables and results and reordered the tables and results.

Accordingly, we revised the manuscript.

The following sentence in the Introduction

“To address this question, we hypothesized that the insulin administration method would be associated with neurological outcomes and changes in blood glucose.”

Was revised as following

“To address this question, we hypothesized that the insulin administration method would be related to blood glucose levels and thus to neurological outcomes.”

The following sentences in the Method

“The primary outcome was neurological outcomes assessed using CPC 6 months after ROSC and defined as good (CPC 1 or 2) or poor (CPC 3–5). The secondary outcomes were maximum glucose, mean glucose, a standard deviation (SD) of glucose, minimum glucose, moderate hypoglycemia, and severe hypoglycemia.”

Was revised as following

“The primary outcome was the achievement of mean blood glucose < 180 mg/dL. The secondary outcomes were neurological outcomes assessed using CPC 6 months after ROSC, maximum glucose, mean glucose, a standard deviation (SD) of glucose, minimum glucose, moderate hypoglycemia, and severe hypoglycemia. The neurological outcomes were defined as good (CPC 1 or 2) or poor (CPC 3–5).”

The following sentence in the Statistical analysis

“We conducted a logistic regression analysis to investigate the association between insulin administration methods and neurological outcomes.”

Was revised as following

“We conducted logistic regression analyses to investigate the association between the insulin administration method and the achievement of mean blood glucose ≤ 180 mg/dL and the association between insulin administration methods and neurological outcomes.”

The following sentences in the Statistical analysis

“All variables with a *p*-value < 0.2 in univariate analyses were included in the multivariate logistic regression analysis to find the covariates. We performed the Hosmer–Lemeshow test to test the goodness-of-fit of the logistic model. We selected age, first monitored rhythm, etiology of cardiac arrest, time from collapse to ROSC, PaO_2_ after ROSC_,_ PaCO_2_ after ROSC, and pre-TTM shock as covariates those had *p*-value < 0.05 in the multivariate logistic regression analysis. We conducted the logistic regression analysis with insulin administration methods and covariates.”

Was revised as following

“We selected the variables with a *p*-value < 0.05 in comparisons among insulin administration methods as covariates for the association between insulin administration methods and mean blood glucose ≤ 180 mg/dL. Additionally, we selected the covariates after performing the multivariable logistic regression analysis with the variables with a p-value < 0.2 in comparisons between groups of mean glucose ≤ 180 mg/dL and > 180 mg/dL. We also selected the covariates for the association between insulin administration methods and neurological outcomes through the multivariable logistic regression analysis with variables with a *p*-value <0.2 in comparisons between neurological outcome groups. We selected the variables with a *p*-value < 0.05 in the multivariate logistic regression analyses as final covariates. We performed the Hosmer–Lemeshow test to test the goodness-of-fit of the logistic model.”

We reordered the Tables and added table 3 and table 4, which compares characteristics between mean glucose ≤ 180 mg/dL and mean glucose > 180 mg/dL and multivariable regression analysis between insulin administration methods and mean glucose ≤ 180 mg/dL

Table 2 🡪 table 1

Table 3 🡪 table 2

Table 1 🡪 table 5

Table 4 🡪 table 6

Table 5 🡪 table 7

Table 6 🡪 table 8

Glucose after ROSC among three insulin administration methods was moved to Table 1.

Mean glucose ≤ 180 mg/dL was added in the table 2 and revised the following sentence in the result.

The following sentence in the Results

“Mean glucose, SD of glucose, and minimum glucose were different among the three groups.”

Was revised as following

“Achievement of mean glucose ≤ 180 mg/dL, Mean glucose, SD of glucose, and minimum glucose differed among the three groups.”

The following tables (Table 3 and Table 4) and paragraphs were added.

“**Association between insulin administration method and mean glucose under 180 mg/dL**

Table 3 shows the demographic and clinical characteristics stratified by mean glucose of 180 mg/dL. A total of 296 (53.9%) achieved mean glucose ≤ 180 mg/dL. Patients who had mean glucose ≤ 180 mg/dL had lower BMI (23.0 kg/m^2^ [20.7–25.4]) vs. 23.9 kg/m^2^ [21.3–26.1]) than those had mean glucose > 180 mg/dL. Patients with mean glucose ≤ 180 mg/dL had fewer pre-existing illnesses (hypertension and diabetes mellitus). Patient who had mean glucose ≤ 180 mg/dL fewer witnesses of collapse (208/296 vs. 154/253), lower epinephrine dose (2 mg [0–4] vs. 2 mg [1–5]), lower serum lactate (9.3 mg/dL [6.0–12.6] vs. 11.3 mg/dL [8.0–14.0]), higher PaO_2_ (117.5 mmHg [76.0–221.0] vs. 100.0 mmHg [71.0–160.0]), lower pre-TTM shock (155/296 vs. 176/253), lower SOFA scores (11 [9–13] vs. 12 [10–14]), and a higher proportion of target temperature of 33℃–34℃ (255/296 vs. 192/253).”

Table 3. Demographic and clinical characteristics stratified by mean glucose of 180 mg/dL

| Variables | Total (n = 549) | MG ≤ 180 mg/dL (n = 296) | MG > 180 mg/dL (n = 253) | *p* |
| --- | --- | --- | --- | --- |
| Age, years | 61.0 (50.0–71.0) | 59.0 (49.0–71.0) | 62.0 (53.5–71.0) | 0.093 |
| Male sex | 369 (67.2) | 199 (67.2) | 170 (67.2) | 1.000 |
| Body mass index, kg/m^2^ | 23.5 (20.9–25.8) | 23.0 (20.7–25.4) | 23.9 (21.3–26.1) | 0.025 |
| Pre-existing illness |  |  |  |  |
| Coronary artery disease | 69 (12.6) | 31 (10.5) | 38 (15.0) | 0.141 |
| Heart failure | 25 (4.6) | 12 (4.1) | 13 (5.1) | 0.688 |
| Hypertension | 242 (44.1) | 112 (37.8) | 130 (51.4) | 0.002 |
| Diabetes mellitus | 195 (35.5) | 63 (21.3) | 132 (52.2) | <0.001 |
| Stroke or TIA | 41 (7.5) | 18 (6.1) | 23 (9.1) | 0.240 |
| Pulmonary disease | 41 (7.5) | 28 (9.5) | 13 (5.1) | 0.079 |
| Renal disease | 45 (8.2) | 25 (8.4) | 20 (7.9) | 0.941 |
| Liver cirrhosis | 5 (0.9) | 2 (0.7) | 3 (1.2) | 0.860 |
| Witnessed | 362 (65.9) | 208 (70.3) | 154 (60.9) | 0.026 |
| Bystander CPR | 338 (61.6) | 185 (62.5) | 153 (60.5) | 0.690 |
| Shockable rhythm | 163 (29.7) | 98 (33.1) | 65 (25.7) | 0.072 |
| Cardiac etiology | 345 (62.8) | 184 (62.2) | 161 (63.6) | 0.789 |
| Time to ROSC, min | 32.0 (19.0–45.0) | 32.0 (19.0–45.0) | 33.0 (19.0–46.0) | 0.365 |
| Epinephrine dose, mg | 2 (1–4) | 2 (0–4) | 2 (1–5) | 0.017 |
| Serum lactate, mg/dL | 10.2 (6.7–13.3) | 9.3 (6.0–12.6) | 11.3 (8.0–14.0) | <0.001 |
| Glucose after ROSC, mg/dL | 287 (228–360) | 263 (215–323) | 323 (242–409) | <0.001 |
| PaO_2,_ mmHg | 106.8 (73.0–191.0) | 117.5 (76.0–221.0) | 100.0 (71.0–160.0) | 0.001 |
| PaCO_2,_ mmHg | 49.6 (37.0–71.4) | 48.3 (37.1–69.5) | 54.0 (38.8–75.0) | 0.343 |
| Pre-TTM shock | 331 (60.3) | 155 (52.4) | 176 (69.6) | <0.001 |
| SOFA | 12 (9–13) | 11 (9 – 13) | 12 (10 – 14) | <0.001 |
| Target temperature |  |  |  | 0.003 |
| 33℃–34℃ | 447 (81.4) | 255 (86.1) | 192 (75.9) |  |
| 35℃–36℃ | 102 (18.6) | 41 (13.9) | 61 (24.1) |  |

MG, mean glucose; TIA, transient ischemic attack; CPR, cardiopulmonary resuscitation; ROSC, return of spontaneous circulation; TTM, targeted temperature management; SOFA, sequential organ failure assessment

“Multivariable logistic regression analysis found hypertension (aOR, 0.609; 95% CIs, 0.399–0.930), diabetes mellitus (aOR, 0.273; 95% CIs, 0.175–0.425), witnessed (aOR, 1.992; 95% CIs, 1.289–3.078), PaO_2_ (aOR, 1.002; 95% CIs, 1.000–1.004), pre-TTM shock (aOR, 0.541; 95% CIs, 0.355–0.826), target temperature of 35–36℃ (aOR, 0.500; 95% CIs, 0.297–0.842), glucose after ROSC (aOR, 0.995; 95% CIs, 0.993–0.997) as covariates. The insulin administration method was not associated with the achievement of mean glucose ≤ 180 mg/dL after adjusting covariates (Table 4).”

**Table 4. The association between insulin administration method and mean glucose ≤ 180 mg/dL**

| Variables | Adjusted odds ratio (95% confidence interval) | *p* |
| --- | --- | --- |
| Hypertension | 0.686 (0.458 – 1.027) | 0.067 |
| Diabetes mellitus | 0.274 (0.180 – 0.417) | < 0.001 |
| Renal disease | 1.559 (0.742 – 3.277) | 0.241 |
| Witnessed | 1.752 (1.156 – 2.656) | 0.008 |
| Cardiac etiology | 0.891 (0.575 – 1.381) | 0.607 |
| Time to ROSC | 0.999 (0.989 – 1.009) | 0.820 |
| Glucose after ROSC, mg/dL | 0.995 (0.993 – 0.997) | <0.001 |
| PaO_2,_ mmHg | 1.003 (1.001 – 1.004) |  |
| PaCO_2,_ mmHg | 1.003 (0.996 – 1.011) | 0.387 |
| Pre-TTM shock | 0.580 (0.389 – 0.864) | 0.007 |
| Target temperature |  |  |
| 33℃–34℃ | Reference |  |
| 35℃–36℃ | 1.819 (1.094 – 3.025) |  |
| Insulin administration method |  |  |
| CII | Reference |  |
| SQI | 0.848 (0.493 – 1.461) | 0.553 |
| IBI | 0.673 (0.415 – 1.091) | 0.108 |

ROSC, return of spontaneous circulation; TTM, targeted temperature management; CII, continuous intravenous insulin; SQI, subcutaneous insulin; IBI, intravenous bolus insulin

Table 4 was revised as Table 6 as following

**Table 6. The association between insulin administration method and poor neurological outcomes**

| Variables | Adjusted odds ratio (95% confidence interval) | *p* |
| --- | --- | --- |
| Age, years | 1.037 (1.015–1.059) | 0.001 |
| Diabetes mellitus | 0.792 (0.427–1.471) | 0.460 |
| Renal disease | 1.469 (0.461–4.680) | 0.515 |
| Witnessed | 0.776 (0.378–1.590) | 0.488 |
| Shockable rhythm | 0.182 (0.099–0.336) | < 0.001 |
| Cardiac etiology | 0.203 (0.083–0.496) | < 0.001 |
| Time to ROSC, min | 1.063 (1.042–1.083) | < 0.001 |
| PaO_2_, mmHg | 1.003 (1.000–1.005) | 0.049 |
| PaCO_2_, mmHg | 1.014 (1.001–1.028) | 0.036 |
| Pre-TTM shock | 1.632 (0.933–2.854) | 0.086 |
| Target temperature | 1.240 (0.533–2.882) | 0.617 |
| 33℃–34℃ | Reference |  |
| 35℃–36℃ |  |  |
| Insulin administration method |  |  |
| CII | Reference |  |
| SQI | 0.660 (0.335–1.301) | 0.230 |
| IBI | 1.757 (0.867–3.560) | 0.118 |

ROSC, return of spontaneous circulation; TTM, targeted temperature management; CII, continuous intravenous insulin; SQI, subcutaneous insulin; IBI, intravenous bolus insulin

The sentences in the Discussion

“This retrospective analysis found that the insulin administration method had no association with the 6-month neurological outcome. The CII group had the lowest mean glucose levels and the IBI group had the highest SD of glucose levels.”

Was revised as following

“This retrospective analysis found that the insulin administration method had no association with the achievement of mean glucose ≤ 180 mg/dL and the 6-month neurological outcome. Nevertheless, the CII group had the lowest mean glucose levels and the IBI group had the highest SD of glucose levels.”

The sentence in the Discussion

“However, SQI or IBI had no association with the 6-month neurological outcomes compared to CII. This discrepancy between blood glucose variables and clinical outcomes according to the insulin administration method warrants further clinical trials.”

Was revised as following

“However, SQI or IBI had no association with the achievement of mean glucose ≤ 180 mg/dL and the 6-month neurological outcomes compared to CII. It might be postulated that SQI or IBI is not inferior to CII regarding controlling hyperglycemia, considering the independent association between high mean glucose and poor neurological outcome.”

The sentence in the Conclusiono

“The insulin administration method was not associated with the 6-month neurological outcomes in OHCA survivors who underwent TTM. The CII method was associated with lower mean glucose and lower SD of glucose rather than the IBI and SQI methods.”

Was revised as following

“The insulin administration method was not associated with the achievement of mean glucose ≤ 180 mg/dL and the 6-month neurological outcomes in OHCA survivors who underwent TTM. The CII method had lower mean glucose and lower SD of glucose rather than the IBI and SQI methods.”

Abstract was revised as following

“We investigated the association of insulin administration method with the achievement of mean glucose ≤ 180 mg/dL and neurological outcomes in out-of-hospital cardiac arrest (OHCA) survivors who had hyperglycemia after the return of spontaneous circulation. From a multicenter prospective registry, we extracted the data of adult OHCA survivors who underwent targeted temperature management (TTM) between 2015 and 2018. Blood glucose levels every 4 h after initiating TTM were obtained for 72 h. We divided insulin administration methods into three categories: subcutaneous (SQI), intravenous bolus (IBI), and continuous intravenous (CII). We calculated the mean glucose and standard deviation (SD) of glucose. The primary outcome was the achievement of mean glucose ≤ 180 mg/dL. The secondary outcomes were the 6-month neurological outcome based on the Cerebral Performance Category (CPC) scale (good, CPC 1–2; poor, CPC 3–5), mean glucose, and SD of glucose. Of the 549 patients, 296 (53.9%) achieved mean glucose ≤ 180 mg/dL, and 438 (79.8%) had poor neurological outcomes, 134 (24.4%), 132 (24.0), and 283 (51.5%) were in the SQI, IBI, and CII groups, respectively. The SQI (adjusted odds ratio [aOR], 0.848; 95% confidence intervals [CIs], 0.493–1.461) and IBI (aOR, 0.673; 95% CIs, 0.415–1.091) groups were not associated with mean glucose ≤ 180 mg/dL and the SQI (aOR, 0.660; 95% CIs, 0.335–1.301) and IBI (aOR, 1.757; 95% CIs, 0.867–3.560) groups were not associated with poor neurological outcomes compared to the CII group. The CII (168 mg/dL [147–202]) group had the lowest mean glucose than the SQI (181 mg/dL [156–218]) and IBI (184 mg/dL [162–216]) groups. The CII (45.0[33.9–63.5]) group had a lower SD of glucose than the IBI (50.8 [39.1–72.0]) group. The insulin administration method was not associated with achieving mean glucose ≤ 180 mg/dL and 6-month neurological outcomes.”

2. Why patient with adequate glucose control with insulin therapy within 24 hours from ROSC were not included?

Answer) We excluded the patient who had no hyperglycemia (>180 mg/dL) within 24 h after ROSC. In other words, we excluded the patients who did not require insulin administration to control the blood glucose. However, the patients who were given insulin to control blood glucose within 24 h after ROSC were included irrespective of adequate glucose control.
